# Dietary calcium and phosphorus levels in lactating Shenxian sows regulate growth performance and bone metabolism in suckling piglets

**DOI:** 10.3389/fvets.2026.1768617

**Published:** 2026-05-08

**Authors:** Yu Li, Jiachun Song, Wenjun Wang, Shang Li, Jixiang Liu, Chunlian Lu, Hongzhan Cao

**Affiliations:** 1 College of Animal Science and Technology, Agricultural University of Hebei, Baoding, Hebei Province, China; 2 College of Animal Science and Technology, Hebei North University, Zhangjiakou, Hebei, China

**Keywords:** bone metabolism, calcium and phosphorus homeostasis, growth performance of suckling piglets, high-yielding Shenxian sows, OPG/RANKL/RANK signaling pathway

## Abstract

**Background:**

Optimal dietary calcium and phosphorus levels are critical for maximizing milk production in highly prolific sow breeds and subsequent bone development in suckling piglets. However, specific nutritional recommendations for the Shenxian sow are lacking.

**Aim:**

This study aimed to evaluate the effects of graded dietary Ca and P levels during lactation on growth performance, mineral metabolism, and the OPG/RANKL/RANK signaling pathway in nursing piglets.

**Methodology:**

Forty-five multiparous Shenxian sows were randomly assigned to one of five dietary treatments in a completely randomized design (n = 9 sows/treatment). Treatments consisted of a control diet (C: 0.63% Ca, 0.31% available P), a low Ca-P diet (L: 0.55% Ca, 0.27% available P), and three high Ca-P diets (H1: 0.71%/0.35%; H2: 0.79%/0.39%; H3: 0.87%/0.43%). Piglet blood samples were collected via anterior vena cava puncture on days 17 and 35 of lactation for serum biochemical and hormonal analysis.

**Results:**

High dietary Ca-P supplementation linearly improved indicators of mineral retention and bone regulation. Compared with the control group, apparent Ca digestibility increased in H1, H2, and H3 groups by 15.18%, 19.86%, and 16.08%, respectively (*P* < 0.05). Serum P and milk Ca concentrations were elevated in all high Ca-P groups (*P* < 0.05). Notably, the OPG/RANKL ratio, a key marker of inhibited bone resorption, increased by 32.73% and 41.82% in the H2 and H3 groups, respectively (*P* < 0.01). While FGF-23 levels increased on Day 17 in the L, H2, and H3 groups, the H2 group exhibited a subsequent 15.05% reduction by Day 35 (*P* < 0.05), suggesting a time dependent normalization of phosphaturic response. The H2 group also demonstrated superior nitrogen efficiency, with a 26.21% reduction in average daily fecal N excretion (*P* < 0.05).

**Conclusion:**

Elevating dietary Ca and P levels to 0.79% and 0.39%, respectively, optimally enhances piglet growth performance by modulating the OPG/RANKL/RANK axis and reducing postnatal bone resorption. These findings establish evidence based nutritional thresholds for high-yielding Shenxian sows. Future research should investigate the long term effects of these mineral levels on sow longevity and subsequent reproductive cycles.

## 1 Introduction

Genetic selection and improved management have substantially increased the reproductive output of modern sow lines (1). However, in high producing lactating sows, the daily export of calcium (Ca) and phosphorus (P) through milk frequently exceeds dietary intake, leading to maternal bone demineralization and compromised skeletal development in suckling piglets (2). For indigenous breeds such as the Shenxian pig characterized by high prolificacy and distinct lactation physiology the precise dietary Ca and P thresholds required to prevent these adverse outcomes remain undefined.

The Shenxian pig is a representative high-yielding Chinese indigenous fatty black breed with early sexual maturity, robust reproductive performance, tolerance to coarse feed, and high disease resistance. Owing to its exceptional prolificacy, it was officially cataloged in the National List of Livestock and Poultry Genetic Resources of China (3, 4). Reproductive research has advanced from phenotypic selection to molecular mechanisms: symmetric teat numbers and backfat thickness of 20–26 mm enhance reproductive efficiency (5), and GWAS have identified 16 SNP loci associated with reproductive traits, highlighting candidate genes such as *TSKU* and *LRRC32* for marker assisted selection (6).

Despite genetic advances, nutritional standards lag behind Shenxian sow productivity. Tokach et al. (7) reported that modern prolific sows nursing large litters may require up to 55.6 g/d total Ca and 25.1 g/d standardized total tract digestible phosphorus (STTD P) values substantially exceeding NRC (63) estimates. Dietary Ca-P imbalance constitutes a critical challenge: inadequate intake predisposes sows to postpartum paralysis, shortens productive lifespan, and compromises piglet skeletal integrity, whereas excessive intake reduces feed digestibility and increases environmental nutrient excretion (8). Lactation imposes immense physiological demands, and milk Ca and P directly govern neonatal bone development, cellular signal transduction, and energy metabolism (9). Ca-P accumulation during nursing exerts a lifelong influence on bone health (10, 11). When dietary supply is insufficient, sows mobilize skeletal reserves to stabilize milk composition, leading to progressive bone demineralization (12). Beyond the skeleton, disrupted Ca-P homeostasis triggers systemic consequences via the calcium-sensing receptor (CaSR) and FGF-23/Klotho axis (13, 14). The dietary Ca/P ratio is as critical as absolute concentrations (15), and Ca and P rank second only to energy and protein in nutritional importance (16). Optimal recommendations vary across genetic lines the Chinese National Standard (GB/T 39235-2020) recommends 0.65% Ca and 0.57% P (17); Sanyuan hybrids perform optimally at 0.55% Ca/0.53% P (18, 19); Large White pigs require 0.8% Ca/0.8% P (20); Ningxiang pigs require 0.83% Ca/0.67% P (21); and Rongchang sows require 0.82% Ca/0.66% P (22).

Dietary interventions modulate intestinal health and nutrient digestibility by reshaping gut microbiota composition. Chen et al. (23) demonstrated that Litsea cubeba essential oil improved apparent digestibility of calcium, crude protein, and ether extract in pigs, accompanied by altered fecal Microbiota. Ca-P imbalances compromise intestinal morphology and perturb gut microbiota (24), and different calcium sources alter intestinal bacterial communities in weaned piglets (25). Optimized Ca-P levels in Ningxiang pigs upregulated intestinal mineral transporters and enriched beneficial microbial taxa (21). These findings underscore that precise mineral formulation enhances nutrient utilization through host and microbiota dependent mechanisms. Calcium also functions as a ubiquitous second messenger in immune signaling. The CaSR links extracellular mineral status to intracellular inflammatory responses. Lee et al. (26) showed that CaSR activates the NLRP3 inflammasome via increased intracellular Ca^2^? and decreased cAMP. Wang et al. (27) further demonstrated that natural compounds attenuate inflammation by modulating NF-κB and NLRP3 inflammasome signaling. In porcine models, CaSR activation alleviates intestinal inflammation and improves barrier function (28, 29), providing a mechanistic framework for how mineral balance may influence the maternal immune microenvironment during lactation.

Despite extensive research on commercial hybrids, data regarding the specific mineral requirements of the Shenxian pig a high fat indigenous breed with distinct lactation physiology remain scarce. The influence of graded dietary Ca and available P levels during lactation on the OPG/RANKL/RANK signaling axis in suckling piglets remains unexplored. Therefore, we hypothesized that elevating dietary Ca and available P would activate the osteoprotegerin mediated protective pathway, suppressing RANKL induced bone resorption and improving growth performance and bone mineralization in suckling piglets.

This study investigated the effects of varying dietary Ca and available P levels based on GB/T 39235-2020 recommendations on growth performance, mineral metabolism, bone turnover markers, and the OPG/RANKL/RANK signaling system in lactating Shenxian sows and their offspring.

## 2 Materials and methods

### 2.1 Trial materials

The compound premix for lactating sows, dicalcium phosphate (CaHPO4), and limestone powder were supplied by Shijiazhuang Dechang Feed Co., Ltd. (Hebei, China). The Shenxian pig is an indigenous Chinese breed characterized by its adaptability to coarse feed, a relatively high carcass lean meat percentage (approximately 41.3%), and robust reproductive performance. This breed was officially included in the China National Catalog of Livestock and Poultry Genetic Resources in 2016.

### 2.2 Experiment design

A total of 45 healthy, high-producing Shenxian sows (parity 3–4, with no fewer than 11 piglets per litter) were selected and randomly assigned to one of five dietary treatments in a completely randomized design. Each treatment consisted of nine replicates, with one sow per replicate. The experimental period lasted 35 days, encompassing the entire lactation phase.

Dietary treatments were formulated based on the calcium and available phosphorus recommendations outlined in the Chinese National Standard Nutrient Requirements of Swine (GB/T 39235-2020). The five experimental diets were as follows: a control diet (Group C) containing 0.63% Ca and 0.31% P; a low Ca-P diet (Group L) containing 0.55% Ca and 0.27% P; and three high Ca-P diets (Groups H1, H2, and H3) containing 0.71% Ca/0.35% P, 0.79% Ca/0.39% P, and 0.87% Ca/0.43% P, respectively. All diets were thoroughly mixed using a mechanical mixer prior to feeding. The detailed ingredient composition and calculated nutrient levels of the basal diets are presented in Table 1.

**Table 1:** Basic diet composition and nutrient level of lactating sows (%).

Items	Content (%)				
Ingredients	Group C	Group L	Group H1	Group H2	Group H3
Corn	60.79	61.12	60.47	60.14	59.82
Wheat bran	16.00	16.00	16.00	16.00	16.00
Soybean meal	15.00	15.00	15.00	15.00	15.00
Rice bran	5.00	5.00	5.00	5.00	5.00
CaHPO4	1.13	0.88	1.37	1.61	1.85
Limestone	1.08	1.00	1.16	1.25	1.33
Premix^a^	1.00	1.00	1.00	1.00	1.00
Total	100.00	100.00	100.00	100.00	100.00
Nutrition levels^b^					
Metabolic energy (MJ/kg)	12.43	12.47	12.38	12.34	12.30
Crude protein	13.56	14.46	13.98	13.73	14.57
Crude fiber	7.13	7.16	7.25	7.17	7.12
Lysine	1.19	1.19	1.19	1.19	1.19
Methionine + Cysteine	0.34	0.34	0.34	0.34	0.34
Threonine	0.41	0.41	0.41	0.41	0.41
Calcium	0.63	0.55	0.71	0.79	0.87
Total phosphorus	0.55	0.52	0.59	0.63	0.67
Available phosphorus	0.31	0.27	0.35	0.39	0.43

^a^Premix provides: VA 4,800 IU; VD3 800 IU; VE 36 mg; VK3 0.8 mg; VB1 0.6 mg; VB2 4.8 mg; VB6 2 mg; VB12 0.008 mg; nicotinic acid 18 mg; pantothenic acid 10 mg; folic acid 3 mg; biotin 0.2 mg; choline chloride 0.48 g; iron 120 mg; copper 16 mg; zinc 112 mg; manganese 20 mg; iodine 0.36 mg; selenium 0.3 mg.

^b^Crude protein, crude fiber, calcium, phosphorus, phytic acid phosphorus are measured values, the rest are calculated values.

On days 17 (mid lactation) and 35 (late lactation), prior to the morning feeding, healthy suckling piglets with similar body weights were randomly selected from each litter for blood collection via anterior vena cava puncture. All animals were housed in closed farrowing pens under standardized environmental conditions.

### 2.3 Feeding and management

Piglets were weighed individually on the day of farrowing (day 0), on day 21, and at weaning (day 35). Prior to the commencement of the trial, the farrowing facility was thoroughly cleaned, disinfected, and equipped with functional heat lamps. Gestating sows were moved into the farrowing house 7 days before their expected parturition date and were fasted during transport. Following parturition, sows were fed three times daily at 07:00, 12:00, and 18:00 h. On the day of farrowing, sows were not offered feed but had *ad libitum* access to fresh drinking water. All neonatal piglets consumed colostrum within 2 h postpartum. Umbilical cords were clipped and disinfected once piglets achieved independent locomotion. At 7 days of age, piglets were introduced to creep feed. All subsequent husbandry practices, including thermal management and vaccination protocols, were performed in accordance with standard farm operating procedures.

### 2.4 Sample collection and measurement

#### 2.4.1 Production performance, performance, productivity

Individual piglet body weights were recorded at birth, on day 21, and at weaning (day 35). Litter weight was also recorded at these time points. Average daily gain (ADG) was calculated using the following formula: ADG (g/d) = (Weaning litter weight in kg – Birth litter weight in kg) × 1,000 / (35 d × Number of weaned piglets).

#### 2.4.2 Feed sample collection and determination

Feed samples were collected from each dietary treatment using the quartering method, with approximately 300 g obtained per group. Samples were sealed and stored at 4 °C pending analysis. Routine feed quality testing was conducted at the College of Animal Science and Technology, Hebei Agricultural University. Feed samples were oven dried at 55 °C for 48 h, ground to pass through a 1 mm sieve, and stored at 4 °C until chemical composition analysis. Crude protein (Method 2001.11), crude fiber (Method 978.10), calcium (Method 927.02), and total phosphorus (Method 965.17) contents were determined in accordance with AOAC International (64) standard procedures.

#### 2.4.3 Collection and determination of fecal and urine samples

At the conclusion of the experiment (days 33–35), total fecal output was collected daily from one sow per replicate using the total collection method. Feces were weighed, thoroughly homogenized, and a representative 300 g aliquot was obtained. This aliquot was divided into two portions: portion A was acidified with 10% hydrochloric acid at a ratio of 10 ml acid per 100 g fresh feces to prevent nitrogen volatilization and was immediately stored at −20 °C for subsequent nitrogen analysis. Portion B was oven-dried at 55 °C for 48 h, ground to pass through a 1 mm sieve, and stored at −20 °C for the determination of routine nutrients, calcium, and phosphorus.

Concurrently, urine was collected quantitatively from the same sows using the total urine collection method. The total urine volume was recorded, and a 5% aliquot was preserved by acidification with 10% dilute sulfuric acid to achieve a pH < 3. Urine samples were stored in brown glass bottles at −20 °C until analysis. The apparent digestibility of nutrients was calculated according to the methods described by Zhang Liying (30) using the following equation: Apparent digestibility (%) of a nutrient in feed = (intake of that nutrient – amount of that nutrient in feces)/intake of that nutrient × 100%, Nitrogen metabolism: Nitrogen intake (NI), Fecal nitrogen (FN), Urine nitrogen (UN), Total nitrogen (TN), Retained nitrogen (RN), Apparent digestibility (AD), Net protein utilization (NPU), Apparent biological value of protein (ABV). NI = Percentage of *N* in feed × average daily total feed weight, FN = Percentage of *N* in feces × average daily total fecal weight, UN = Percentage of *N* in urine × average daily total urine volume, TN = FN + UN, RN = NI-FN-UN, Apparent digestibility of nitrogen (%) = (NI – FN)/NI × 100%, NPU = RN/NI × 100%, ABV = RN/(NI – FN) × 100%.

#### 2.4.4 Milk collection and measurement

Following parturition, approximately 5 ml of milk was manually collected from the anterior, middle, and posterior mammary glands of each sow. Samples were pooled, transferred to sterile centrifuge tubes, and immediately stored at −20 °C. Concentrations of milk calcium, phosphorus, protein, lactose, and milk fat were quantified using commercial enzyme linked immunosorbent assay (ELISA) kits. All milk analyses were performed by Jiangsu Enzyme Immunoassay Co., Ltd. (Jiangsu, China) according to the manufacturer's specifications.

#### 2.4.5 Blood sample collection and measurement

On days 17 and 35 of lactation, healthy suckling piglets with comparable body weights were randomly selected from each litter. Blood samples (approximately 10 ml) were collected from the anterior vena cava after an overnight fast. Samples were allowed to clot at room temperature and subsequently centrifuged at 3,500 × g for 15 min at 4 °C. The resulting serum was aliquoted into labeled sterile centrifuge tubes and stored at −20 °C until analysis. Serum concentrations of the following parameters were measured using commercial porcine-specific ELISA kits (Jiangsu Enzyme Immunoassay Co, Ltd, Jiangsu, China) strictly following the manufacturer's protocols: calcium, phosphorus, 25-hydroxyvitamin D3 (25(OH)D3), parathyroid hormone (PTH), calcitonin (CT), fibroblast growth factor 23 (FGF-23), estradiol (E_2_), bone morphogenetic protein-2 (BMP-2), sclerostin (SOST), bone-specific alkaline phosphatase (BALP), total alkaline phosphatase (ALP), N-terminal telopeptide of type I collagen (NTX-I), C-terminal telopeptide of type I collagen (CTX-I), tartrate-resistant acid phosphatase 5b (TRACP-5b), pyridinoline (PYD), cathepsin K (CTSK), receptor activator of nuclear factor-κB ligand (RANKL), receptor activator of nuclear factor-κB (RANK), and osteoprotegerin (OPG).

### 2.5 Statistical analysis

Data were initially organized using Microsoft Excel 2019. Prior to formal statistical testing, all data were screened for normality using the Shapiro-Wilk test and for homogeneity of variances using Levene's test. For variables that satisfied both assumptions, one-way analysis of variance (ANOVA) was conducted, followed by Duncan's multiple range test for *post-hoc* comparisons among treatment means. When the assumption of homogeneity of variances was violated, Welch's ANOVA was employed with the Games-Howell *post-hoc* test. For data that were not normally distributed, the non-parametric Kruskal–Wallis test was applied, and Dunn's test was used for multiple comparisons. The individual sow was considered the experimental unit for all statistical analyses (*n* = 9 per treatment group). All statistical computations were performed using SPSS Statistics version 25.0 (IBM Corp., Armonk, NY, USA). Data are presented as means ± standard deviation (SD). Differences were considered statistically significant at *P* < 0.05. Pearson correlation analysis among serum biomarkers was conducted using OriginPro software (Version 2021, OriginLab Corporation, Northampton, MA, USA).

## 3 Results

### 3.1 Effects of different calcium and phosphorus levels on nutrient apparent digestibility in lactating sows

The ingredient composition of the five experimental diets (Groups C, L, H1, H2, and H3) was consistent, comprising corn, wheat bran, soybean meal, rice bran, calcium hydrogen phosphate, limestone powder, and a vitamin-mineral premix. The apparent digestibility of nutrients, determined from fecal samples collected during days 33–35 of lactation, is presented in Table 2. No significant differences were observed among the five groups for the apparent digestibility of crude protein (*P* = 0.561), crude ash (*P* = 0.102), total energy (*P* = 0.807), or dry matter (*P* = 0.103). Compared with Group C, the apparent digestibility of crude fat in Group L was significantly higher by 6.87% (*P* = 0.039), whereas the apparent digestibility of phosphorus in Group L was significantly lower by 13.72% (*P* < 0.01). No significant differences in crude fat or phosphorus digestibility were detected between the high-calcium-phosphorus groups (H1, H2, H3) and Group C (*P* > 0.05). However, the apparent digestibility of calcium in Groups H1, H2, and H3 was significantly increased by 15.18%, 19.86%, and 16.08%, respectively, relative to Group C (*P* = 0.010).

**Table 2:** Effects of different calcium and phosphorus levels on apparent digestibility of nutrients in lactating sows (%).

Items	Group C	Group L	Group H1	Group H2	Group H3	*P*-value
CP	82.24 ± 1.62	82.42 ± 2.96	81.14 ± 3.09	80.17 ± 6.41	80.97 ± 2.91	0.561
EE	75.16 ± 4.05^b^	80.32 ± 4.48^a^	76.20 ± 3.79^ab^	75.26 ± 3.01^b^	79.15 ± 3.74^ab^	0.039
Ash	37.79 ± 2.22	35.52 ± 2.61	37.24 ± 1.73	36.67 ± 3.50	35.07 ± 2.50	0.102
GE	81.67 ± 1.79	80.36 ± 3.29	79.96 ± 4.07	79.91 ± 2.99	81.08 ± 2.28	0.807
Ca	50.06 ± 8.44^b^	55.44 ± 5.67^ab^	57.66 ± 3.18^a^	60.00 ± 6.69^a^	58.11 ± 7.88^a^	0.010
P	45.85 ± 2.58^a^	40.32 ± 2.24^b^	43.58 ± 3.15^a^	44.77 ± 2.73^a^	45.09 ± 3.04^a^	< 0.01
DM	80.14 ± 1.54	78.59 ± 2.72	78.50 ± 2.45	77.43 ± 2.59	78.89 ± 1.86	0.103

^1^In same row, different superscript letters indicate significant difference (*P* < 0.05), and same superscript letters or no letters indicate no significant difference (*P* > 0.05).The calcium content in the Group C is 0.63%, and the available phosphorus content is 0.31%; the calcium content in Experimental Group L is 0.55%, and the available phosphorus content is 0.27%; the calcium content in Experimental Group H1 is 0.71%, and the available phosphorus content is 0.35%; the calcium content in Experimental Group H2 is 0.79%, and the available phosphorus content is 0.39%; the calcium content in Experimental Group H3 is 0.87%, and the available phosphorus content is 0.43%.

### 3.2 Effects of different calcium and phosphorus levels on nitrogen metabolism in lactating sows

Nitrogen balance parameters are summarized in Table 3. No significant differences were detected among groups for nitrogen intake (*P* = 0.383), urinary nitrogen excretion (*P* = 0.293), total nitrogen excretion (*P* = 0.053), retained nitrogen (*P* = 0.558), or net protein utilization (*P* = 0.150). Compared with Group C, average daily fecal nitrogen excretion in Group H2 was significantly reduced by 26.21% (*P* < 0.01), and apparent nitrogen digestibility was significantly increased by 3.86% (*P* = 0.006). No other treatment groups differed significantly from Group C for these parameters (*P* > 0.05).

**Table 3:** Effects of different calcium and phosphorus levels on nitrogen metabolism in lactating sows.

Items	Group C	Group L	Group H1	Group H2	Group H3	*P*-value
NI (g/d)	54.70 ± 6.84	51.67 ± 7.12	52.85 ± 10.72	48.52 ± 2.21	50.97 ± 9.73	0.383
FN (g/d)	10.30 ± 1.67^a^	9.57 ± 1.67^a^	9.44 ± 1.39^a^	7.60 ± 0.28^b^	9.94 ± 1.65^a^	< 0.01
UN (g/d)	19.23 ± 3.42	18.26 ± 1.38	19.54 ± 3.43	18.01 ± 0.65	19.92 ± 2.34	0.293
TN (g/d)	29.53 ± 5.03	27.83 ± 2.92	28.97 ± 4.57	25.61 ± 0.76	29.86 ± 3.77	0.053
RN (g/d)	25.17 ± 3.33	23.84 ± 6.50	23.88 ± 7.51	22.92 ± 2.41	21.11 ± 6.53	0.558
AD (%)	81.18 ± 1.61^b^	81.34 ± 3.14^b^	81.72 ± 3.24^ab^	84.31 ± 2.56^a^	80.24 ± 3.04^b^	0.006
NPU (%)	46.13 ± 4.09	45.50 ± 7.49	44.23 ± 7.82	47.13 ± 2.88	40.66 ± 5.88	0.150

^1^In same row, different superscript letters indicate significant difference (*P* < 0.05), and same superscript letters or no letters indicate no significant difference (*P* > 0.05).The calcium content in the Group C is 0.63%, and the available phosphorus content is 0.31%; the calcium content in Experimental Group L is 0.55%, and the available phosphorus content is 0.27%; the calcium content in Experimental Group H1 is 0.71%, and the available phosphorus content is 0.35%; the calcium content in Experimental Group H2 is 0.79%, and the available phosphorus content is 0.39%; the calcium content in Experimental Group H3 is 0.87%, and the available phosphorus content is 0.43%.

### 3.3 Effects of different calcium and phosphorus levels on milk composition of lactating sows

Milk composition data are shown in Table 4. No significant differences were observed among the five groups for colostrum lactoprotein (*P* = 0.271), colostrum lactose (*P* = 0.097), mature milk lactose (*P* = 0.112), colostrum butterfat (*P* = 0.351), mature milk butterfat (*P* = 0.082), colostrum milk calcium (*P* = 0.680), colostrum milk phosphorus (*P* = 0.462), or mature milk phosphorus (*P* = 0.261). Mature milk lactoprotein content was highest in Group H2. Compared with Groups L, H1, and H3, mature milk lactoprotein content in Group H2 was significantly increased by 28.32%, 29.52%, and 29.28%, respectively (*P* = 0.041). Dietary calcium and phosphorus levels exerted a significant effect on mature milk calcium concentration (*P* < 0.01). Mature milk calcium content increased progressively with elevated dietary calcium and phosphorus levels, with Groups H1, H2, and H3 exhibiting significant increases of 7.89%, 8.10%, and 21.4%, respectively, compared with Group C (*P* < 0.01).

**Table 4:** Effects of different levels of calcium and phosphorus on milk composition of lactating sows.

Items	Type	Group C	Group L	Group H1	Group H2	Group H3	*P*-value
Lactoprotein (mg/ml)	Colostrum	24.48 ± 2.37	20.65 ± 2.84	22.13 ± 3.42	24.64 ± 1.11	23.67 ± 4.29	0.271
	Maturemilk	24.98 ± 3.23^ab^	21.64 ± 5.49^b^	21.44 ± 2.13^b^	27.77 ± 0.82^a^	21.48 ± 1.95^b^	0.041
Lactose (mg/ml)	Colostrum	4.08 ± 0.07	4.35 ± 0.02	3.74 ± 0.90	3.86 ± 0.53	3.70 ± 0.10	0.097
	Maturemilk	3.84 ± 0.03	3.87 ± 0.02	3.95 ± 0.09	3.89 ± 0.03	3.87 ± 0.07	0.112
Butterfat (mg/ml)	Colostrum	31.97 ± 4.82	24.08 ± 3.98	26.65 ± 7.73	30.60 ± 7.55	28.46 ± 2.74	0.351
	Maturemilk	28.41 ± 0.79	31.21 ± 2.79	29.54 ± 1.34	28.88 ± 0.90	30.84 ± 0.74	0.082
MilkCalcium (nmol/L)	Colostrum	16.44 ± 2.79	16.65 ± 2.65	18.51 ± 2.91	18.75 ± 1.63	19.06 ± 1.47	0.680
	Maturemilk	14.69 ± 0.26^c^	13.52 ± 0.15^d^	15.85 ± 0.62^b^	15.88 ± 0.24^b^	17.83 ± 0.44^a^	< 0.01
MilkPhosphorus (nmol/L)	Colostrum	4.83 ± 1.50	4.79 ± 0.27	5.59 ± 0.36	5.14 ± 1.24	3.81 ± 0.13	0.462
	Maturemilk	4.83 ± 1.34	4.97 ± 0.23	5.20 ± 0.92	5.93 ± 0.19	5.62 ± 0.35	0.261

^1^In same row, different superscript letters indicate significant difference (*P* < 0.05), and same superscript letters or no letters indicate no significant difference (*P* > 0.05).The calcium content in the Group C is 0.63%, and the available phosphorus content is 0.31%; the calcium content in Experimental Group L is 0.55%, and the available phosphorus content is 0.27%; the calcium content in Experimental Group H1 is 0.71%, and the available phosphorus content is 0.35%; the calcium content in Experimental Group H2 is 0.79%, and the available phosphorus content is 0.39%; the calcium content in Experimental Group H3 is 0.87%, and the available phosphorus content is 0.43%.

### 3.4 Effects of different levels of calcium and phosphorus on growth performance of lactating piglets

Growth performance data are presented in Table 5. No significant differences were detected among treatment groups for average birth weight (*P* = 0.514), 21-day average weight (*P* = 0.121), average birth litter weight (*P* = 0.973), 21-day litter weight (*P* = 0.387), weaning litter weight (*P* = 0.065), or average daily gain (*P* = 0.091). Notably, although ADG did not reach the conventional significance threshold, weaning weight was significantly affected by dietary treatment. Compared with Group H2, weaning weights in Groups C, L, H1, and H3 were significantly lower by 10.67%, 16.52%, 8.64%, and 9.28%, respectively (*P* < 0.01). The ADG *P*-value of 0.091, while not meeting the α = 0.05 threshold, indicates a clear numerical trend toward improved growth in Group H2 (193.15 vs. 169.49 g/d in Group C, a relative increase of approximately 14%). This numerical advantage in daily gain, when accumulated over the 35-day lactation period, translated into a statistically significant difference in weaning weight.

**Table 5:** Effects of different levels of calcium and phosphorus on growth performance of suckling piglets.

Items	Group C	Group L	Group H1	Group H2	Group H3	*P*-value
ALW (kg)	1.05 ± 0.23	1.08 ± 0.20	1.03 ± 0.21	1.08 ± 0.22	1.06 ± 0.25	0.514
21 AW (kg)	4.36 ± 0.72	4.16 ± 1.14	4.41 ± 1.19	4.55 ± 1.04	4.45 ± 0.89	0.121
AWW (kg)	7.03 ± 1.01^bc^	6.57 ± 1.46^c^	7.19 ± 1.70^b^	7.87 ± 1.82^a^	7.14 ± 1.19^b^	< 0.01
AILW (kg)	12.20 ± 3.43	12.61 ± 2.20	12.07 ± 3.39	11.85 ± 1.63	12.56 ± 2.76	0.973
21 ALW (kg)	41.17 ± 8.05	42.55 ± 12.69	48.72 ± 13.36	44.00 ± 8.31	49.00 ± 8.48	0.387
AWLW (kg)	64.82 ± 15.85	62.09 ± 13.84	78.27 ± 17.05	73.47 ± 10.67	77.77 ± 13.73	0.065
ADG (g/d)	169.49 ± 22.91	157.53 ± 26.87	177.47 ± 32.31	193.15 ± 28.21	174.99 ± 14.51	0.091

^1^In same row, different superscript letters indicate significant difference (*P* < 0.05), and same superscript letters or no letters indicate no significant difference (*P* > 0.05).The calcium content in the Group C is 0.63%, and the available phosphorus content is 0.31%; the calcium content in Experimental Group L is 0.55%, and the available phosphorus content is 0.27%; the calcium content in Experimental Group H1 is 0.71%, and the available phosphorus content is 0.35%; the calcium content in Experimental Group H2 is 0.79%, and the available phosphorus content is 0.39%; the calcium content in Experimental Group H3 is 0.87%, and the available phosphorus content is 0.43%.

### 3.5 Effects of different calcium and phosphorus levels on serum biochemical indexes of weaned piglets

Serum calcium and phosphorus concentrations at mid and late lactation are presented in Table 6. At mid lactation, no significant difference was observed for serum calcium among groups (*P* = 0.078), whereas serum phosphorus differed significantly (*P* < 0.01). Compared with Group C, serum phosphorus concentrations in Groups H1, H2, and H3 were significantly elevated by 38.98%, 24.45%, and 40.43%, respectively (*P* < 0.01). At late lactation, serum calcium did not differ significantly among groups (*P* = 0.363), but serum phosphorus remained significantly affected by dietary treatment (*P* < 0.01). Serum phosphorus in Groups H1, H2, and H3 was significantly higher than in Groups C and L (*P* < 0.01).

**Table 6:** Effects of different calcium and phosphorus levels on serum biochemical indexes of suckling piglets.

Items	Period	Group C	Group L	Group H1	Group H2	Group H3	*P*-value
Ca (mmol/L)	ML	15.33 ± 2.04	13.70 ± 1.57	17.38 ± 4.20	18.58 ± 0.23	18.72 ± 0.33	0.078
	LL	16.98 ± 2.13	15.20 ± 1.82	18.17 ± 3.77	19.11 ± 0.50	18.54 ± 2.71	0.363
P (mmol/L)	ML	4.13 ± 0.15^c^	3.87 ± 0.21^c^	5.74 ± 0.34^a^	5.14 ± 0.36^b^	5.80 ± 0.08^a^	< 0.01
	LL	4.66 ± 0.06^b^	3.82 ± 0.18^c^	5.31 ± 0.60^a^	5.49 ± 0.10^a^	5.02 ± 0.16^ab^	< 0.01

^1^In same row, different superscript letters indicate significant difference (*P* < 0.05), and same superscript letters or no letters indicate no significant difference (*P* > 0.05).The calcium content in the Group C is 0.63%, and the available phosphorus content is 0.31%; the calcium content in Experimental Group L is 0.55%, and the available phosphorus content is 0.27%; the calcium content in Experimental Group H1 is 0.71%, and the available phosphorus content is 0.35%; the calcium content in Experimental Group H2 is 0.79%, and the available phosphorus content is 0.39%; the calcium content in Experimental Group H3 is 0.87%, and the available phosphorus content is 0.43%.

### 3.6 Effects of different calcium and phosphorus levels on serum hormones in weaned piglets

Serum hormone concentrations are summarized in Table 7. At mid lactation no significant differences were observed among groups for 25-hydroxyvitamin D3 (*P* = 0.321), parathyroid hormone (*P* = 0.287), or sclerostin (*P* = 0.207). However, compared with Group C, calcitonin concentrations in Groups H1, H2, and H3 were significantly increased by 10.16%, 12.36%, and 21.63%, respectively (*P* < 0.01). Fibroblast growth factor 23 concentrations in Groups L, H2, and H3 were significantly elevated by 26.67%, 17.77%, and 31.24%, respectively (*P* < 0.01). Estradiol concentration in Group H3 was significantly decreased by 16.02% (*P* < 0.01), whereas bone morphogenetic protein-2 was significantly increased by 17.07% (*P* = 0.005).

**Table 7:** Effects of different calcium and phosphorus levels on serum hormones in suckling piglets.

Items	Period	Group C	Group L	Group H1	Group H2	Group H3	*P*-value
25HVD3 (μg/L)	ML	13.58 ± 1.82	15.26 ± 0.19	14.03 ± 1.38	13.56 ± 1.57	13.71 ± 0.52	0.321
	LL	14.44 ± 0.26	12.87 ± 1.40	13.25 ± 0.06	13.07 ± 1.59	13.02 ± 2.18	0.510
PTH (ng/L)	ML	140.91 ± 13.60	149.61 ± 2.84	148.42 ± 15.66	148.42 ± 7.73	158.94 ± 9.95	0.287
	LL	150.58 ± 1.47^a^	152.50 ± 4.21^a^	136.14 ± 8.86^b^	137.73 ± 1.81^b^	128.00 ± 1.93^c^	< 0.01
CT (ng/L)	ML	113.16 ± 1.05^c^	104.15 ± 2.45^d^	124.66 ± 1.86^b^	127.15 ± 1.88^b^	137.64 ± 4.53^a^	< 0.01
	LL	108.17 ± 6.22	109.79 ± 3.68	112.25 ± 7.72	113.10 ± 10.83	114.04 ± 7.74	0.800
FGF-23 (pg/ml)	ML	485.39 ± 12.41^c^	614.84 ± 10.94^a^	511.41 ± 54.48^c^	571.65 ± 5.93^b^	637.07 ± 26.37^a^	< 0.01
	LL	592.37 ± 12.04^ab^	589.59 ± 47.23^ab^	603.67 ± 59.92^a^	503.17 ± 27.77^c^	537.84 ± 22.32^bc^	< 0.01
E (pmol/L)	ML	1,689.33 ± 164.94^ab^	1,790.13 ± 44.18^a^	1,713.86 ± 39.97^ab^	1,546.08 ± 157.78^bc^	1,418.69 ± 77.27^c^	< 0.01
	LL	1,730.82 ± 146.53	1,753.54 ± 104.18	1,514.82 ± 152.79	1,621.28 ± 136.12	1,535.30 ± 132.20	0.078
BMP-2 (ng/L)	ML	129.85 ± 15.43^b^	128.40 ± 2.73^b^	140.11 ± 5.34^ab^	125.33 ± 11.59^b^	152.02 ± 2.83^a^	0.005
	LL	128.74 ± 6.83	129.35 ± 20.67	134.00 ± 4.39	136.03 ± 5.37	135.76 ± 10.67	0.816
SOST (pg/ml)	ML	243.09 ± 10.50	250.46 ± 40.86	237.18 ± 12.19	212.97 ± 5.32	246.38 ± 25.67	0.207
	LL	273.24 ± 22.28	284.81 ± 1.34	262.38 ± 16.26	246.46 ± 33.98	247.31 ± 10.23	0.069

^1^In same row, different superscript letters indicate significant difference (*P* < 0.05), and same superscript letters or no letters indicate no significant difference (*P* > 0.05).The calcium content in the Group C is 0.63%, and the available phosphorus content is 0.31%; the calcium content in Experimental Group L is 0.55%, and the available phosphorus content is 0.27%; the calcium content in Experimental Group H1 is 0.71%, and the available phosphorus content is 0.35%; the calcium content in Experimental Group H2 is 0.79%, and the available phosphorus content is 0.39%; the calcium content in Experimental Group H3 is 0.87%, and the available phosphorus content is 0.43%.

At late lactation, the previously observed differences in calcitonin (*P* = 0.800), estradiol (*P* = 0.078), and BMP-2 (*P* = 0.816) were no longer statistically significant. No significant differences were detected for 25(OH)D3 (*P* = 0.510) or sclerostin (*P* = 0.069) among groups at this time point. Compared with Group C, PTH concentrations in Groups H1, H2, and H3 were significantly decreased by 9.5%, 8.5%, and 14.9%, respectively (*P* < 0.01). FGF-23 concentration in Group H2 was significantly reduced by 15.05% relative to Group C (*P* < 0.01).

### 3.7 The impact of different levels of calcium and phosphorus on bone formation markers in suckling piglets

Bone formation marker data are presented in Table 8. Bone specific alkaline phosphatase activity did not differ significantly among groups at either mid lactation (*P* = 0.094) or late-lactation (*P* = 0.423). Total alkaline phosphatase activity was significantly affected by dietary treatment at both time points (*P* < 0.01). At mid-lactation, compared with Group C, ALP activity in Group H2 was significantly decreased by 10.36% (*P* < 0.01), whereas Group L exhibited a significant increase of 9.89% (*P* < 0.01). At late lactation, ALP activity in Group L remained significantly elevated by 5.26% (*P* < 0.01), while Group H3 showed a significant decrease of 9.85% (*P* < 0.01) compared with Group C.

**Table 8:** Effects of different levels of calcium and phosphorus on bone formation markers in suckling piglets.

Items	Period	Group C	Group L	Group H1	Group H2	Group H3	*P*-value
BAP (IU/L)	ML	363.41 ± 46.06	359.83 ± 6.55	340.54 ± 42.38	346.45 ± 57.05	357.46 ± 13.42	0.094
	LL	355.67 ± 24.63	387.66 ± 13.13	335.92 ± 37.26	354.34 ± 45.27	343.56 ± 57.90	0.423
AKP (U/L)	ML	46.22 ± 0.72^b^	50.79 ± 0.42^a^	45.97 ± 3.51^b^	41.43 ± 4.55^c^	41.74 ± 0.85^bc^	< 0.01
	LL	43.54 ± 1.27^ab^	45.83 ± 2.05^a^	43.83 ± 0.85^ab^	41.41 ± 2.58^bc^	39.25 ± 0.98^c^	< 0.01

^1^In same row, different superscript letters indicate significant difference (*P* < 0.05), and same superscript letters or no letters indicate no significant difference (*P* > 0.05).The calcium content in the Group C is 0.63%, and the available phosphorus content is 0.31%; the calcium content in Experimental Group L is 0.55%, and the available phosphorus content is 0.27%; the calcium content in Experimental Group H1 is 0.71%, and the available phosphorus content is 0.35%; the calcium content in Experimental Group H2 is 0.79%, and the available phosphorus content is 0.39%; the calcium content in Experimental Group H3 is 0.87%, and the available phosphorus content is 0.43%.

### 3.8 The impact of different calcium and phosphorus levels on bone resorption markers in weaned piglets

Bone resorption marker data are summarized in Table 9. At mid lactation, no significant differences were observed among groups for NTX-I (*P* = 0.082), TRACP-5b (*P* = 0.064), or PYD (*P* = 0.520). Compared with Group C, CTX-I concentrations in Groups H1 and H2 were significantly increased by 11.18% and 8.46%, respectively (*P* = 0.040). Cathepsin K activity in Groups H2 and H3 was significantly decreased by 6.60% and 15.77%, respectively (*P* < 0.01).

**Table 9:** Effects of different calcium and phosphorus levels on bone resorption markers in suckling piglets.

Items	Period	Group C	Group L	Group H1	Group H2	Group H3	*P*-value
NTX-I (μg/L)	ML	10.13 ± 0.14	10.09 ± 0.18	9.44 ± 1.34	10.21 ± 0.99	8.79 ± 0.25	0.082
	LL	10.32 ± 0.15^a^	10.75 ± 0.41^a^	9.90 ± 0.25^a^	10.29 ± 0.35^a^	8.94 ± 1.05^b^	< 0.01
CTX-I (μg/L)	ML	16.55 ± 0.52^b^	17.88 ± 0.35^ab^	18.40 ± 1.13^a^	17.95 ± 0.26^a^	17.36 ± 1.14^ab^	0.040
	LL	17.54 ± 1.80^a^	17.02 ± 0.37^ab^	16.75 ± 0.50^ab^	15.42 ± 0.76^b^	15.77 ± 0.61^b^	0.036
TRACP5b (U/L)	ML	72.81 ± 1.37	75.44 ± 1.50	72.83 ± 1.52	71.19 ± 0.32	66.85 ± 8.08	0.064
	LL	67.99 ± 3.05	71.18 ± 1.31	61.29 ± 4.61	66.22 ± 8.02	66.83 ± 8.65	0.254
PYD (nmol/L)	ML	53.22 ± 7.45	55.27 ± 0.76	52.83 ± 7.08	56.54 ± 4.57	50.33 ± 2.99	0.520
	LL	56.07 ± 1.51^ab^	60.72 ± 1.33^a^	58.03 ± 3.67^a^	56.10 ± 9.47^ab^	49.20 ± 1.65^b^	0.039
CTS-K (IU/L)	ML	149.48 ± 3.95^b^	161.64 ± 5.06^a^	146.75 ± 1.81^bc^	139.62 ± 8.59^c^	125.90 ± 1.23^d^	< 0.01
	LL	155.22 ± 9.09^a^	149.75 ± 9.28^a^	152.05 ± 13.19^a^	140.39 ± 8.33^ab^	127.51 ± 2.79^b^	< 0.01

^1^In same row, different superscript letters indicate significant difference (*P* < 0.05), and same superscript letters or no letters indicate no significant difference (*P* > 0.05).The calcium content in the Group C is 0.63%, and the available phosphorus content is 0.31%; the calcium content in Experimental Group L is 0.55%, and the available phosphorus content is 0.27%; the calcium content in Experimental Group H1 is 0.71%, and the available phosphorus content is 0.35%; the calcium content in Experimental Group H2 is 0.79%, and the available phosphorus content is 0.39%; the calcium content in Experimental Group H3 is 0.87%, and the available phosphorus content is 0.43%.

At late lactation, TRACP-5b activity did not differ significantly among groups (*P* = 0.254). However, compared with Group C, CTX-I concentrations in Groups H2 and H3 were significantly reduced by 12.08% and 10.09%, respectively (*P* = 0.036). CTSK activity in Group H2 was significantly decreased by 9.55% (*P* < 0.01), and NTX-I concentration in Group H3 was significantly reduced by 13.37% (*P* < 0.01). PYD concentration in Group H3 was significantly lower than in Groups L and H1 (*P* = 0.039).

### 3.9 Effects of varying calcium and phosphorus levels on the OPG-RANKL-RANK signaling pathway in weaned piglets

As shown in Table 10, at mid lactation, no significant differences were detected among groups for serum OPG content (*P* = 0.124), RANKL (*P* = 0.566), RANK (*P* = 0.246), the OPG/RANKL ratio (*P* = 0.274), or the RANK/RANKL ratio (*P* = 0.423). Although OPG concentrations in the high-Ca-P groups were numerically elevated relative to Group C at this time point, these differences did not reach statistical significance. At late lactation, however, OPG content was significantly affected by dietary treatment (*P* < 0.01). Compared with Group C, OPG concentrations in Groups H1, H2, and H3 were significantly increased by 10.36%, 18.88%, and 28.52%, respectively (*P* < 0.05). Similarly, the OPG/RANKL ratio in Groups H2 and H3 was significantly elevated by 32.73% and 41.82%, respectively, relative to Group C (*P* < 0.01).

**Table 10:** Effects of different calcium and phosphorus levels on OPG-RANKL-RANK signal system of suckling piglets.

Items	Period	Group C	Group L	Group H1	Group H2	Group H3	*P*-value
OPG (pg/ml)	ML	1,940.39 ± 138.65	1,837.37 ± 158.31	1,992.37 ± 361.66	2,040.73 ± 137.90	2,229.35 ± 23.03	0.124
	LL	1,759.53 ± 103.98^d^	1,740.37 ± 49.13^d^	1,941.99 ± 38.17^c^	2,091.77 ± 49.81^b^	2,261.37 ± 26.92^a^	< 0.01
RANKL (pmol/L)	ML	789.47 ± 19.81	726.81 ± 15.11	775.78 ± 79.39	776.52 ± 87.17	731.01 ± 87.62	0.566
	LL	766.54 ± 112.29	753.79 ± 49.83	780.62 ± 35.36	684.99 ± 22.41	692.07 ± 23.78	0.114
RANK (pg/ml)	ML	752.76 ± 141.21	775.59 ± 19.47	790.25 ± 6.50	781.50 ± 59.57	681.47 ± 40.58	0.246
	LL	791.17 ± 75.55	858.80 ± 27.17	696.62 ± 33.34	755.44 ± 141.23	747.98 ± 55.54	0.101
OPG/RANKL	ML	0.59 ± 0.05	0.60 ± 0.06	0.62 ± 0.18	0.63 ± 0.03	0.73 ± 0.09	0.274
	LL	0.55 ± 0.04^b^	0.55 ± 0.05^b^	0.59 ± 0.04^b^	0.73 ± 0.02^a^	0.78 ± 0.02^a^	< 0.01
RANK/RANKL	ML	0.23 ± 0.05	0.25 ± 0.01	0.24 ± 0.02	0.24 ± 0.01	0.22 ± 0.01	0.423
	LL	0.25 ± 0.01	0.27 ± 0.02	0.22 ± 0.02	0.26 ± 0.05	0.26 ± 0.03	0.113

^1^In same row, different superscript letters indicate significant difference (*P* < 0.05), and same superscript letters or no letters indicate no significant difference (*P* > 0.05).The calcium content in the Group C is 0.63%, and the available phosphorus content is 0.31%; the calcium content in Experimental Group L is 0.55%, and the available phosphorus content is 0.27%; the calcium content in Experimental Group H1 is 0.71%, and the available phosphorus content is 0.35%; the calcium content in Experimental Group H2 is 0.79%, and the available phosphorus content is 0.39%; the calcium content in Experimental Group H3 is 0.87%, and the available phosphorus content is 0.43%.

### 3.10 Pearson correlation analysis of serum indicators in weaned piglets under different calcium and phosphorus levels

#### 3.10.1 Effects of different calcium and phosphorus levels on serum index correlation of midterm sows piglets

**Figure 1:**
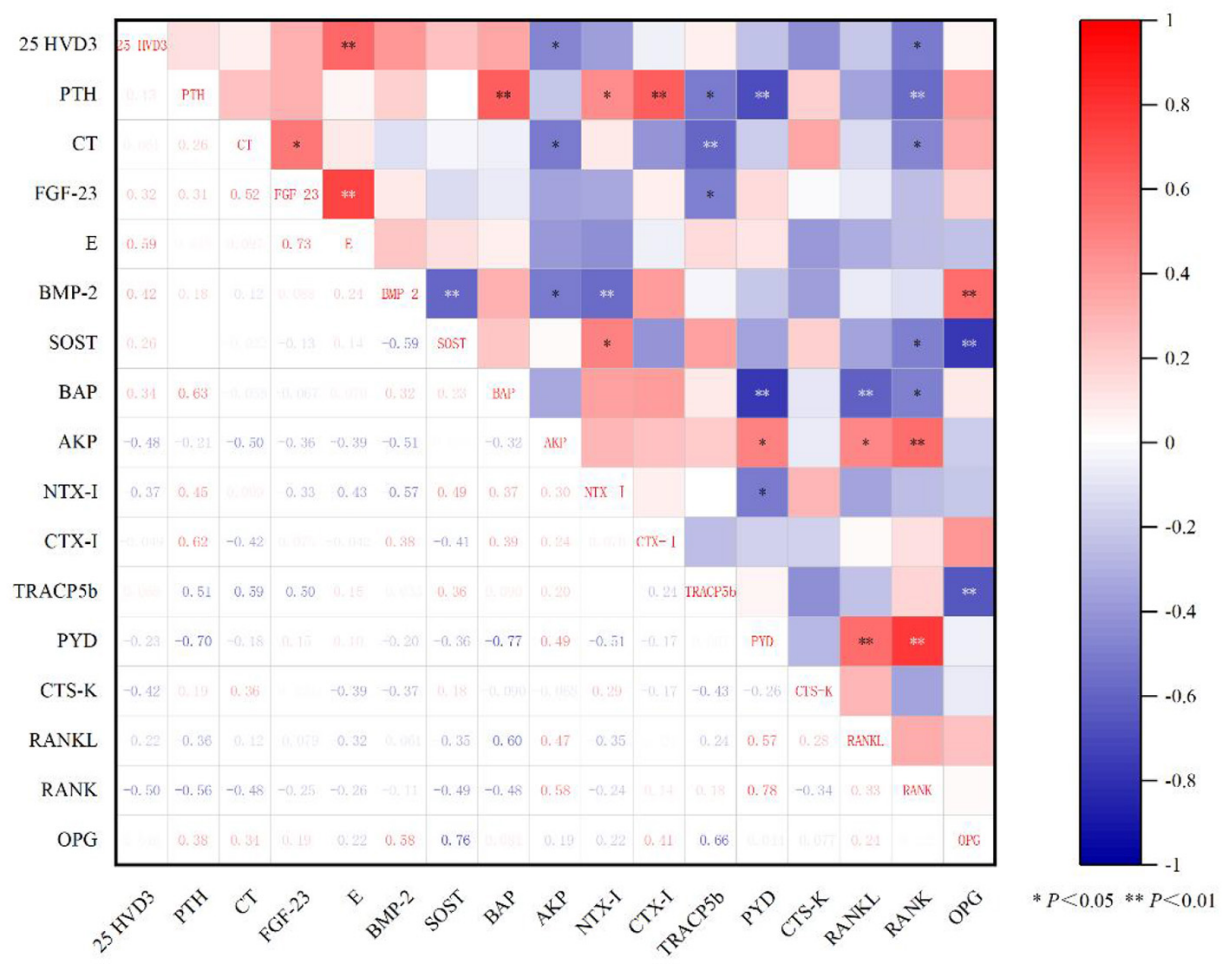
Pearson correlation analysis of different calcium and phosphorus levels on serum indexes of mid-lactation piglets.

As can be seen from Figure 1, 25HVD3 is extremely significantly positively correlated with E (*r* = 0.59, *P* < 0.01), but significantly negatively correlated with AKP and RANK (*r* = −0.48, *r* = −0.50, *P* < 0.05). CT was negatively correlated with AKP, RANK and TRACP5b (*r* = −0.50, *r* = −0.48, *r* = −0.59, *P* < 0.05). Among the bone metabolism regulatory factors, BMP-2 was extremely significantly positively correlated with OPG (*r* = 0.58, *P* < 0.01), while SOST was extremely significantly negatively correlated with OPG (*r* = −0.76, *P* < 0.01). In addition, BAP was extremely significantly negatively correlated with PYD and RANKL (*r* = −0.77, *r* = −0.60; *P* < 0.01), and significantly negatively correlated with RANK (*r* = −0.48, *P* < 0.05). AKP was significantly positively correlated with PYD, RANKL, and RANK (*r* = 0.49, *r* = 0.47; *r* = 0.58, *P* < 0.05). TRACP5b was extremely significantly negatively correlated with OPG (*r* = −0.66, *P* < 0.01).

#### 3.10.2 Effects of different calcium and phosphorus levels on serum index correlation of late sow pigs

**Figure 2:**
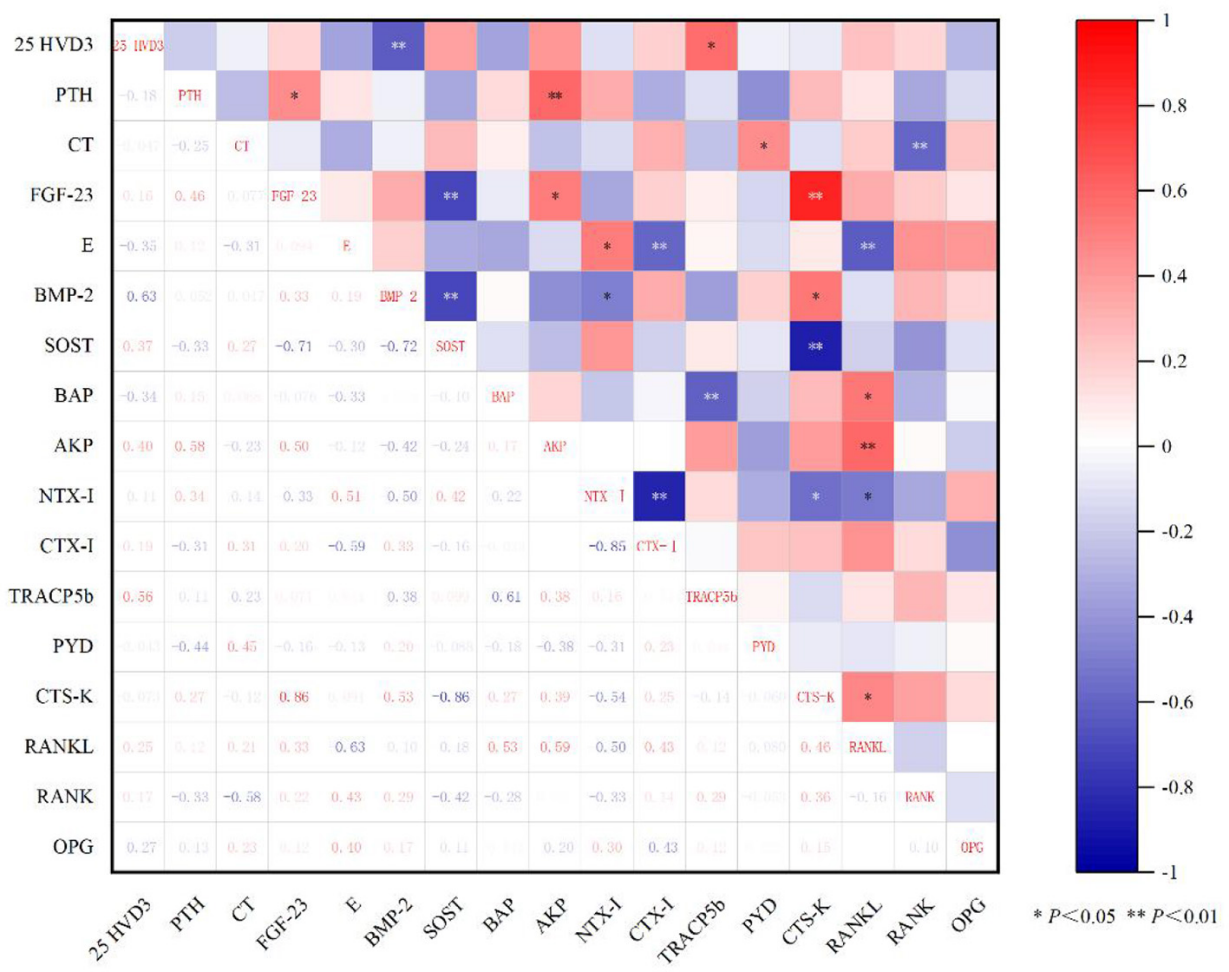
Pearson correlation analysis of different calcium and phosphorus levels on serum indexes of end-lactation piglets.

It can be seen from Figure 2 that 25HVD3 is highly positively correlated with BMP-2 (*r* = 0.63, *P* < 0.01). PTH was positively correlated with AKP (*r* = 0.58, *P* < 0.01) and FGF-23 (*r* = 0.46, *P* < 0.05). FGF-23 was positively correlated with CTS-K (*r* = 0.86, *P* < 0.01), and positively correlated with AKP (*r* = 0.50, *P* < 0.05). SOST was significantly negatively correlated with CTS-K (*r*= −0.86, *P* < 0.01). CTS-K was significantly positively correlated with RANKL (*r* = 0.46, *P* < 0.05).

## 4 Discussion

### 4.1 Effects of different calcium and phosphorus levels on nutrient apparent digestibility and nitrogen metabolism in lactating sows

The apparent digestibility of nutrients in lactating sows is a critical determinant of overall production efficiency. The lack of significant dietary effects on the digestibility of major nutrient fractions observed herein suggests that, within the ranges evaluated, calcium and phosphorus levels do not compromise the digestive utilization of these dietary components. This finding aligns with Shipman et al. (31) who reported no effect of Ca and P on crude protein digestibility, and with Zhai et al. (32) who observed unchanged gross energy digestibility following phytase supplementation. The progressive increase in calcium and phosphorus digestibility with elevated dietary Ca-P levels can be attributed to several interconnected physiological mechanisms. First, adequate dietary calcium upregulates intestinal Calbindin-D9k via VDR-dependent activation, facilitating transcellular calcium transport (33). Second, the optimized Ca:P ratio likely minimized insoluble calcium phytate complexes; excessive inorganic phosphorus acts as an antinutritional factor by precipitating divalent cations (34). Third, the CaSR modulates paracellular mineral permeability in response to luminal Ca^2^?, providing additional homeostatic control (29). Regarding nitrogen metabolism, nitrogen excretion and retention did not differ significantly among groups, suggesting independent regulation of N and P homeostasis within the tested ranges. Notably, Group H2 exhibited the lowest fecal nitrogen output and highest apparent nitrogen digestibility, indicating that optimal Ca-P supply may enhance protein utilization by maintaining gastrointestinal integrity (35). This is consistent with Liang et al. (36) who reported decreased fecal nitrogen with reduced dietary phosphorus. Collectively, these findings underscore that precise Ca-P formulation mitigates nitrogen wastage without compromising mineral retention.

### 4.2 Effects of different calcium and phosphorus levels on milk composition of lactating sows

Colostrum and mature milk serve as the primary sources of energy, immunoglobulins, and minerals for neonatal piglets, and their composition is dynamically influenced by maternal nutritional status (37, 38). In this study, elevating dietary Ca and P levels resulted in a dose-dependent increase in mature milk calcium concentration, with Group H3 exhibiting a 21.4% elevation relative to the control group. Milk protein content was maximized in Group H2, whereas milk phosphorus and lactose remained largely unaffected.

The differential response of milk components to dietary mineral manipulation likely reflects species-specific regulatory mechanisms (39). Qi et al. (40) reported linear increases in both milk phosphorus and protein in sows following Ca-P supplementation, whereas studies in dairy cattle have yielded contrasting results. Although cross species comparisons require caution, parallels can be drawn with findings in poultry, where optimized nutrient intake has been shown to improve product quality via mitigation of oxidative stress (41). This suggests that the enhanced milk composition observed in the present study may not solely reflect direct mineral transfer, but could also involve indirect improvements in maternal metabolic and antioxidant status a hypothesis that warrants further investigation.

### 4.3 Effects of different calcium and phosphorus levels on growth performance of lactating piglets

In the present study, piglet body weight and ADG did not differ significantly during the first 21 days of lactation, consistent with the capacity of sows to mobilize skeletal reserves to buffer short-term deficiencies (42). However, by weaning, Group H2 piglets exhibited significantly greater body weights, confirming that sustained adequate Ca-P supply confers cumulative growth advantages. The absence of further improvement in Group H3 likely reflects the formation of insoluble calcium phosphate precipitates at supra-optimal Ca intakes (43).

Beyond direct nutritional effects, nutrient supply interacts with intestinal development and immune function to drive neonatal growth. He et al. (44) demonstrated that IUGR piglets exhibit impaired ileal immunity and reduced microbial α-diversity, underscoring the centrality of immune competence and gut ecology to neonatal growth. Although we did not directly assess these parameters, the superior performance of Group H2 may partly reflect enhanced barrier function and reduced inflammation secondary to optimized mineral homeostasis. This interpretation aligns with evidence that farm level management and biosecurity practices significantly influence productivity outcomes highlighting the multifactorial nature of growth in commercial settings (45).

At the cellular level, the CaSR links extracellular Ca^2^? to intracellular signaling cascades (46). In lactating mammals, mammary CaSR senses circulating calcium and modulates PTHrP secretion and calcium transport into milk (47), providing a mechanistic framework for how dietary Ca-P supply translates into physiological outcomes.

### 4.4 Effects of different calcium and phosphorus levels on calcium and phosphorus metabolism in lactating piglets

Maternal dietary Ca and P directly modulate milk mineral bioavailability, thereby influencing mineral metabolism in suckling offspring (48). In this study, serum phosphorus was elevated in high Ca-P groups at both time points, whereas serum calcium remained stable reflecting tight homeostatic control. At mid-lactation, CT was elevated in all high Ca-P groups, consistent with its role in restraining osteoclast mediated resorption. Concurrently, FGF-23 was elevated in Groups L, H2, and H3, representing an appropriate phosphaturic response to increased dietary phosphorus load (10). By late lactation, FGF-23 in Group H2 declined significantly while serum phosphorus remained high, suggesting successful establishment of a new homeostatic set point. The re-elevation of FGF-23 in Group H3 may indicate homeostatic overload (49). PTH was suppressed in all high Ca-P groups at late lactation, confirming that maternal supply met neonatal requirements without necessitating PTH mediated resorption. The balanced PTH–FGF-23–CT profile in Group H2 exemplifies ideal mineral homeostasis.

### 4.5 Bone Metabolism and the OPG-RANKL-RANK Signaling Pathway

Calcium and phosphorus are indispensable for skeletal development in neonatal piglets (50). In this study, BALP activity did not differ among groups, whereas total ALP was significantly reduced in Group H2 at mid-lactation. Because ALP activity typically declines as the skeleton reaches functional maturity, this reduction coupled with superior growth suggests that bone development in Group H2 had progressed to a more mature, mineralized state (51, 52). At mid lactation, CTX-I was elevated in Groups H1 and H2, while CTSK was suppressed in Groups H2 and H3, indicating uncoupled remodeling wherein resorptive machinery is partially activated yet degradative capacity is restrained (53, 54). By late lactation, both CTX-I and CTSK were reduced in Group H2, signifying global suppression of resorption as the skeleton transitioned toward an anabolic state (55).

The OPG/RANKL/RANK axis constitutes the final common pathway regulating osteoclastogenesis (56). The OPG/RANKL ratio serves as a quantitative index of osteoclastogenic potential (57). In this study, OPG/RANKL ratios in Groups H2 and H3 at late lactation increased by 32.73% and 41.82%, respectively. A 30%−40% elevation in this ratio is predicted to reduce osteoclast numbers and resorptive activity by 30%−50% (58, 59), effectively uncoupling resorption from the high calcium demand of lactation and preserving trabecular microarchitecture. The preferential elevation in H2 and H3 underscores the existence of a dietary Ca-P threshold required to fully engage this protective cascade (60).

### 4.6 Effects of different calcium and phosphorus levels on the correlations of serum indicators in lactating pigs

Pearson correlation analysis revealed distinct patterns of endocrine and metabolic coordination at mid vs. late lactation. At mid lactation, serum 25(OH)D3 was positively correlated with estradiol and negatively correlated with ALP and RANK, suggesting that adequate vitamin D status, in concert with estrogenic signaling, contributes to the suppression of osteoclastogenesis during this developmental window (61). The positive correlation between BMP-2 and OPG, coupled with the negative correlation between SOST and OPG, defines a pro formation anti resorption regulatory axis that likely operates to maximize net bone accrual. Furthermore, the negative correlations between bone formation markers (BALP) and multiple resorption markers (PYD, RANKL) indicate that bone formation and resorption are tightly coupled in a reciprocal manner during mid-lactation.

At late lactation, the correlation landscape shifted toward a resorption-dominant phenotype. 25(OH)D3 remained positively correlated with BMP-2, indicating that the vitamin D–BMP-2 signaling axis is maintained even as the overall metabolic milieu favors bone resorption, possibly as a preparatory mechanism for post-weaning skeletal recovery (62). PTH was positively correlated with both ALP and FGF-23, underscoring its central role in driving bone resorption while simultaneously coordinating phosphorus excretion via FGF-23 to prevent hyperphosphatemia. The strong positive correlation between FGF-23 and CTSK (*r* = 0.86, *P* < 0.01) provides direct statistical evidence linking the phosphotropic hormone FGF-23 to osteoclast mediated bone resorption, a relationship that is further reinforced by the positive correlation between FGF-23 and ALP. Conversely, the robust negative correlation between SOST and CTSK (*r* = −0.86, *P* < 0.01) suggests that SOST may exert a restraining influence on osteoclast function, potentially serving as a protective feedback mechanism to limit excessive bone loss during the catabolic phase of late lactation.

## 5 Conclusions

In conclusion, elevating dietary calcium and available phosphorus levels to 0.79% and 0.39%, respectively, optimally enhances suckling piglet growth performance by suppressing bone resorption through activation of the OPG/RANKL/RANK signaling axis and by stabilizing systemic mineral homeostasis via coordinated regulation of PTH and FGF-23. These findings establish evidence based mineral recommendations specifically tailored to high-yielding Shenxian sows, addressing a critical gap in breed specific nutritional standards. However, this study is limited by its focus on a single lactation cycle and the absence of direct intestinal or immune assessments. Future research should investigate the long term effects of these dietary Ca-P levels on sow longevity and reproductive performance across successive parities.

## Data Availability

The raw data supporting the conclusions of this article will be made available by the authors, without undue reservation.
